# p53 -Dependent and -Independent Nucleolar Stress Responses 

**DOI:** 10.3390/cells1040774

**Published:** 2012-10-15

**Authors:** Karl Holmberg Olausson, Monica Nistér, Mikael S. Lindström

**Affiliations:** Department of Oncology-Pathology, Karolinska Institutet, Cancer Center Karolinska R8:05, Karolinska University Hospital in Solna, SE-17176, Stockholm, Sweden; Email: Karl.Holmberg.Olausson@ki.se (K.H.O.); Monica.Nister@ki.se (M.N.)

**Keywords:** nucleolus, p53, ribosome biogenesis, cancer, RPL11

## Abstract

The nucleolus has emerged as a cellular stress sensor and key regulator of p53-dependent and -independent stress responses. A variety of abnormal metabolic conditions, cytotoxic compounds, and physical insults induce alterations in nucleolar structure and function, a situation known as nucleolar or ribosomal stress. Ribosomal proteins, including RPL11 and RPL5, become increasingly bound to the p53 regulatory protein MDM2 following nucleolar stress. Ribosomal protein binding to MDM2 blocks its E3 ligase function leading to stabilization and activation of p53. In this review we focus on a number of novel regulators of the RPL5/RPL11-MDM2-p53 complex including PICT1 (GLTSCR2), MYBBP1A, PML and NEDD8. p53-independent pathways mediating the nucleolar stress response are also emerging and in particular the negative control that RPL11 exerts on Myc oncoprotein is of importance, given the role of Myc as a master regulator of ribosome biogenesis. We also briefly discuss the potential of chemotherapeutic drugs that specifically target RNA polymerase I to induce nucleolar stress.

## 1. Introduction

Nucleoli are dynamic structures described a few hundred years ago as distinct nuclear compartments, easily visualized under the light microscope, and the place of birth for ribosomes [1]. High resolution mass spectrometry techniques have revealed that the human genome encodes around 4,500 proteins with potential for nucleolar localization [2,3]. In addition, quantitative proteomics shows that the nucleolar proteome is not static but changes upon different growth conditions or cellular stress [4]. For instance, live cell imaging studies of ribosomal proteins fused to green fluorescent protein illustrate that they are in a state of dynamic exchange, rapidly shuttling between the nucleolus and nucleoplasm [5]. The exchange of proteins and RNA is likely to be facilitated by the lack of a classical lipid bi-layer membrane around the nucleolus.

Nucleoli form around ribosomal RNA (rRNA)-coding chromosomal repeats upon initiation of transcription by RNA polymerase I. One human nucleolus may contain around 400 copies of the rDNA gene encoding 18S and 28S mature rRNA, although not all of the copies are actively transcribed at a given moment [6]. The rDNA is organized in the form of tandemly arranged repeats at the chromosomal nucleolar organizer regions (NORs) [7]. The nucleolus assembles around transcription and maturation of the rRNA, and can be divided into three major subcompartments; fibrillar centers (FCs), dense fibrillar component (DFC) and granular component (GC). Transcription of the rDNA repeats is thought to occur at the border between the FC and DFC, with most RNA polymerase I (RNA pol I) subunits located in the FC region [7,8]. The 28S, 18S and 5.8S ribosomal RNAs (rRNAs) are transcribed by RNA pol I as a single 47S precursor that is subsequently processed and cleaved. The various rRNA species are also post-transcriptionally modified through interaction with small nucleolar ribonucleoproteins (snoRNPs) and additional processing factors in the DFC and GC regions. Ribosome subunit assembly and rRNA processing continues in the GC region, which is especially rich in RNA and protein, while additional processing and modification steps also occur throughout the nucleoplasm [7,8]. The ribosome subunits interact with the nuclear export machinery and are transported, in an inactive state, to the cytoplasm [7,8]. In the cytoplasm several additional maturation steps occur in both the 40S and 60S biogenesis pathways including removal of inhibitory factors for translation ultimately culminating in the functional activation of the ribosome [9].

The nucleolus has traditionally been viewed as a factory for building ribosomes and indeed many of the now identified nucleolar proteins have defined functions in ribosome biogenesis. However, in the first proteomic screen of the nucleolus around 30% of the identified nucleolar proteins were previously unknown [3,10]. Several nucleolar proteins that are not considered to be involved in ribosome biogenesis were also identified [10]. In fact, it has become more and more evident over the last two decades that the nucleolus takes part in the regulation of multiple cellular functions, such as control of the cell-cycle apparatus, ageing, cellular stress responses, mRNA export and modification, protein degradation and sequestration. Nucleoli also play a role in the maturation, assembly and export of RNP (ribonucleoprotein) particles including the signal recognition particle, telomerase RNP, and in processing of pre-tRNAs and U6 snRNA [7,11,12,13,14,15]. Furthermore, the emerging role of the nucleolus as an organizing center for different chromosomal domains within the cell nucleus may have far reaching implications for our understanding of epigenetic and genetic regulation of the genome [16,17,18].

## 2. p53 and Nucleolar Stress

Synthesis of new ribosomes is an essential and energy consuming cellular process controlled directly or indirectly by oncogenes and tumor suppressors including c-Myc, PTEN and p53 [19]. p53 is the principal guardian of the genome and of the cell itself, preventing the initiation of cancer and its progression [20]. The tumor suppressor p53 is activated by a broad range of cellular stressors, including DNA damage, oncogene activation, hypoxia, metabolic errors, heat shock, and nucleolar stress [21,22,23]. p53 transactivates a set of target genes that inhibit cell cycle progression, facilitate DNA repair, induce apoptosis or autophagy. Other target genes of p53 are involved in induction of senescence or differentiation, thus p53 is a master regulator of cell fate [22,23]. In turn, p53 is tightly controlled by the MDM2 oncoprotein that targets p53 for nuclear export and proteasomal degradation [21,22,23]. In fact, MDM2 is the main E3 ubiquitin ligase for p53 and loss of its function is invariably embryonic lethal in mice due to illegitimate induction of p53-dependent apoptosis [24]. Under normal conditions, p53 is maintained at a very low level, but when nucleoplasmic levels of p53 increase as a consequence of for instance DNA damage, this leads to the activation and repression of a large number of genes. The increase in p53 levels is often a result of disrupted MDM2-p53 binding, an event triggered by multiple mechanisms including phosphorylation and/or binding of regulatory proteins [21]. 

**Figure 1:**
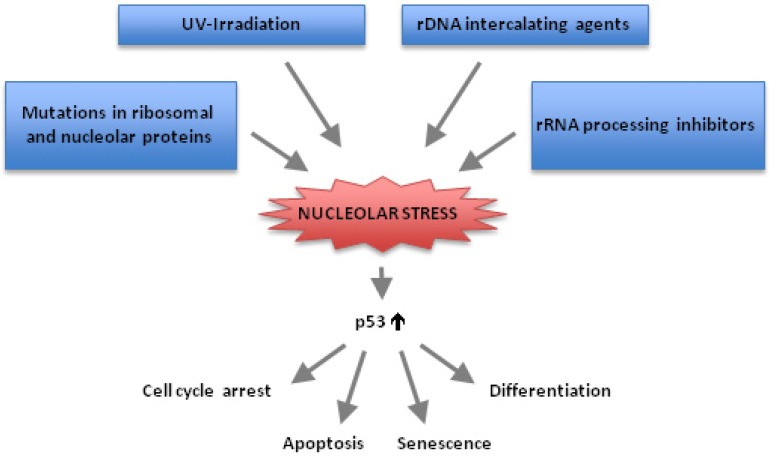
Conditions that induce nucleolar stress and lead to p53-induced effects on cells. A wide range of cell stressors, small molecules, or mutant nucleolar/ribosomal proteins can block ribosomal RNA synthesis or processing thereby inhibiting ribosome biogenesis. The resulting nucleolar stress activates signaling pathways that may lead to p53-induced cell cycle arrest, apoptosis, differentiation, and/or senescence dependent on cell type and level of stress.

The nucleolus responds quickly to several forms of cellular stress (Figure 1) [11,25,26] in both p53-dependent and -independent ways. In a seminal paper from 2001, Pestov and co-workers described how the expression of dominant negative mutants of the nucleolar protein Bop1 resulted in a block in ribosome biogenesis that was followed by p53-induced cell cycle arrest [27]. It is now known that a variety of chemotherapeutic drugs, UV-irradiation and mutant nucleolar/ribosomal proteins result in disturbed nucleolar function and impaired ribosome biogenesis (Figure 1) [28,29]. Impaired or faulty ribosome biogenesis elicits a p53-dependent cellular stress response referred to as “nucleolar stress” or “ribosomal stress” [15]. The term ribosomal stress takes into consideration that not all abnormalities in ribosome biogenesis involve a visible breakdown of the nucleolus. For instance, defects in ribosome biogenesis may occur in nuclear export of ribosome subunits. Disruption of ribosome biogenesis and/or the nucleolar structure activates p53-dependent signaling pathways that may lead to cell cycle arrest [30], apoptosis [31], differentiation [32] or senescence [33,34]. Nucleolar stress also activates p53-independent pathways as will be discussed.

## 3. Chemotherapeutic Drugs Often Block Ribosome Biogenesis and Induce Nucleolar Stress

One bottleneck in cancer cell proliferation is the rate of ribosome production since an abundance of ribosomes is needed to sustain cell growth and proliferation. HeLa cells for instance produce several thousands of ribosomes per minute [35]. It is therefore not surprising that many anti-cancer drugs interfere with RNA pol I or RNA pol II transcription leading to preferential targeting of dividing cancer cells. Interestingly, many commonly used chemotherapeutic drugs first and foremost inhibit rRNA synthesis and/or processing [28]. Examples of these compounds are low concentrations of Actinomycin D [36], 5-Fluorouracil (5-FU) [37] and Mycophenolic acid [38]. Actinomycin D is an antibiotic compound and DNA intercalating molecule [39]. Although Actinomycin D (trade name: Cosmegen) is a potent inhibitor of cell proliferation its use in cancer chemotherapy is limited to a few cancer types including Wilm´s tumor [40], several types of sarcoma for example Ewing´s sarcoma [41], and gestational trophoblastic tumors [42]. Actinomycin D has been widely used in the experimental studies of the nucleolus [43]. At low concentrations (<10nM), the drug preferentially inhibits the production of rRNA by intercalating into the GC rich regions of rDNA to inhibit Pol I-mediated transcription of nascent 47S rRNA [44,45]. The effect of Actinomycin D on the nucleolus is strikingly potent, within an hour after exposure cells exhibit shrinkage and re-organization of the nucleoli. This is accompanied by a massive and sudden release of proteins from the nucleolus into the nucleoplasm followed shortly thereafter by a rapid increase in p53 protein levels. 

Many chemotherapeutic drugs inhibit either rDNA transcription or rRNA processing [28] but so far the contribution of these particular effects over the general DNA damage response/block of DNA replication in restraining overall cancer cell growth remains unclear. Perhaps the effect of inhibition of ribosome biogenesis is more important than previously considered? Small molecule compounds that specifically inhibit rDNA transcription have recently been developed, e.g. CX-3543 (quarfloxin) that inhibits elongation of RNA pol I transcription and with clinical benefit in neuroendocrine tumors [34]. CX-5461 is an inhibitor of RNA pol I transcription that impairs binding of SL1/TIF-1B to the rDNA promoter [33]. This compound selectively kills B-cell lymphoma cells *in vivo*, but not normal B cells, by inducing a p53-dependent apoptotic program activated by nucleolar disruption [46]. The type of non-genotoxic activation of p53 represented by the CX-5461 holds great promise in future cancer therapy. Whether selective targeting of ribosome biogenesis will be of broad clinical value in anti-cancer treatment remains to be seen. Unfortunately, a majority of human cancers have lost wild type p53 functions, but recent results indicate that nucleolar stress also results in cell cycle arrest independently of p53 function by other specific mechanisms, including degradation of the E2F-1 transcription factor [47].

## 4. Changes In Nucleolar Morphology Following Cellular Stress

**Figure 2:**
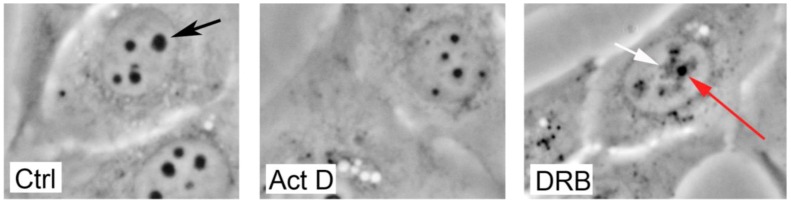
Examples of changes in nucleolar morphology induced by nucleolar stress. U2OS osteosarcoma cells were treated with 5nM Actinomycin D (Act D) for two hours, which induced a rapid shrinkage of the nucleoli (middle panel) compared to control cells (left panel). The arrow points at one nucleolus in the control sample. U2OS cells were also treated with 5,6-dichloro-1-beta-D-ribofuranosylbenzimidazole (DRB) for 6 hours, a drug that induces unraveling and fragmentation of nucleoli by inhibition of RNA pol II dependent transcription (right panel). The granular component (GC) appears as a remnant dark dot (red arrow) whereas the fibrillar centers containing the rDNA genes unravel and disperse throughout the nucleoplasm surrounding the remnant GC (white arrow).

The nucleolus undergoes dramatic visible morphological changes and molecular re-arrangements when exposed to different cytostatic/cytotoxic compounds [25]. One of the most fascinating alterations that can be seen is perhaps the unraveling and fragmentation of the nucleolus occurring upon inhibition of RNA pol II with the drug DRB (5,6-dichloro-1-beta-D-ribofuranosylbenzimidazole) [43]. DRB primarily inhibits RNA pol II, and presumably as a consequence of the nucleolar fragmentation it also inhibits rRNA processing, but does not prevent rDNA transcription [28]. In cells exposed to DRB, the rDNA within the FCs unravels, extends and decondenses to form a structure known as the “nucleolar necklace” [43,48,49]. The nucleolar necklace can be visualized with antibodies directed against the nucleolar protein fibrillarin (DFC marker) or RNA pol I (FC marker). The disrupted nucleolar structure can also be observed using phase contrast microscopy where the residual GC structure appears as dense dark dots separated from the unraveled FC and DFC regions (Figure 2). 

Another dramatic effect on the nucleolus can be seen in cells exposed to low concentrations of Actinomycin D (<10 nM), that preferentially inhibits transcription by RNA pol I [44]. Actinomycin D induces an easily visible decrease in the nucleolar size (Figure 2), separation of the FC and GC regions, in parallel with the occasional formation of so-called nucleolar caps [43,50]. A change in nucleolar structure can also be seen in cells exposed to inhibitors of rRNA processing such as 5-fluorouracil [28] and a wide variety of other compounds. Knockdown of ribosomal proteins often results in nucleolar stress, but since rDNA transcription in most cases continues, the nucleolus remains intact [51]. However, re-organization in nucleolar chromatin may occur in cells depleted of ribosomal proteins [52]. Thus it appears that p53 is not stabilized by a completely disrupted nucleolus *per se* but rather by a defective nucleolar function [25]. One critical common function may be a defective biogenesis of 18S and 28S rRNA [53], but perhaps it is the entire process of ribosome biogenesis that is monitored by p53? If so, then we should expect to see p53 activation caused by defects in the very early stages of ribosome biogenesis (import of ribosomal proteins into the nucleus) or in the later stages of ribosome biogenesis (export of ribosome subunits). Indeed, this seems to be the case as depletion of importin 7 and exportin 1, proteins that are involved in nuclear import of ribosomal proteins and export of ribosomal subunits, respectively, triggers p53 activation [54].

## 5. Early Evidence From Mouse Models Reveal That Ribosome Biogenesis Defects Activate p53

It is important to emphasize that the majority of ribosomal proteins play critical roles in either ribosomal RNA processing, ribosome subunit assembly or pre-ribosome subunit export [52,55,56,57]. This, and the fact that ribosome biogenesis in itself is such an essential process in the cell, suggests that it is subject to tight regulation and surveillance. The first strong evidence that a checkpoint could be operating to monitor the fidelity of ribosome biogenesis in mammalian cells came from a study using liver-specific inducible deletion of Rps6 in mice, which leads to a deficiency in production of new 40S ribosome subunits [58]. The study revealed that conditional deletion of Rps6 after hepatectomy, resulted in the loss of the regenerative capacity (meaning an increase in cell number), which was due to induction of cell cycle arrest, but not impaired cell growth (size) of the already existing liver cells [58]. Mounting experimental evidence from a number of mouse models has now revealed the existence of a p53 checkpoint sensing the integrity of ribosome biogenesis. For example, deletion of one allele of the ribosomal protein gene Rps6 disrupts ribosome biogenesis, but the early embryonic lethality seen in this model is due to activation of p53-dependent cell cycle arrest and apoptosis [59], rather than to a general decrease in mRNA translation—the major function of mature ribosomes in the cytoplasm. Another study showed that loss of Rps6 negatively affected T-cell accumulation in the spleen and lymph nodes due to p53 activation [60]. Furthermore, mutations in the genes encoding Rps19 and Rps20 in mice result in p53-dependent pigmentation defects (known as epidermal melanocytosis), reduced body size and impaired development of the hematopoietic system [61]. The Belly Spot and Tail (Bst) mouse phenotype is caused by a heterozygous mutation in the Rpl24 gene leading to congenital malformations of the eye and skeleton (a kinked tail), in addition to skin pigmentation abnormalities. The phenotypic defects seen in Bst mice are caused, in most part, by p53 [62]. Surprisingly, deletion of one allele of p53 reverses the Bst mouse phenotype whereas loss of both p53 alleles is lethal, revealing a pro-survival function of low levels of p53 [62]. It is important to mention that not only defects in ribosomal proteins elicit a p53 response, but that this is also the case for other proteins involved in rRNA processing. For instance, inactivation of the nucleolar rRNA processing protein Rbm19 in mice results in p53 activation at the embryonic stage [63]. 

## 6. Human Ribosomopathies—the Rle of p53

An intact ribosome biogenesis apparatus is required for normal human development, and several human genetic disorders that stem from defects in key proteins of the ribosome biogenesis machinery have been described, reviewed in references [64,65]. These disorders are now known as “ribosomopathies”, in which the primary pathophysiology is related to impaired mature ribosome function and/or ribosome biogenesis [64,65]. Similar as in mice, the various defects in ribosome biogenesis present with a surprisingly divergent spectrum of phenotypes in humans. This disease group includes (but is not limited to) Diamond-Blackfan anemia (DBA), a congenital bone marrow failure syndrome, and the 5q-syndrome, a subtype of the myelodysplastic syndrome. Haplo-insufficiency for ribosomal protein genes has been implicated in DBA and the 5q-syndrome. DBA is caused by mutations in ribosomal proteins and is characterized by defective erythropoiesis (hypoplastic macrocytic anemia) and various congenital malformations. RPS19 is to date the most frequently mutated ribosomal protein gene in DBA [66] but mutations have also been found in RPS7, RPS17, RPS24, RPL5, RPL11, RPL26 and RPL35A [67,68,69]. Recently, mutations in a transcription factor GATA1 was found linked to DBA suggesting that not only mutations in ribosomal protein genes result in DBA [70]. The 5q-syndrome is caused by a somatically acquired deletion of chromosome 5q, which leads to haploinsufficiency of RPS14 and an erythroid phenotype similar to DBA [71]. 

The work in mice showing that insufficiency in ribosomal proteins triggers p53 activation suggested that p53 could be involved in mediating some of the phenotypic manifestations of the ribosomopathies in humans. Several mouse models of DBA and 5q-syndromes have been generated, confirming that the p53 pathway is activated via ribosome dysfunction, reviewed in reference [72]. As mentioned, mice with mutations in *Rps19* have pigmentation defects (including hyperpigmented foot pads), decreased body size, and anemia [61]. Crossing the *Rps19* mutant mice with mice lacking p53 rescued the skin and hematopoietic phenotypes [61]. A conditional heterozygous deletion of *Rps14*, in mouse hematopoietic cells, causes macrocytic anemia that can be rescued by loss of p53 [73]. Moreover, conditional inactivation of *Rps6* in mice recapitulates some features of the 5q- syndrome and DBA, in that the mice develop macrocytic anemia and erythroid hypoplasia, and p53 plays a critical role in manifesting these phenotypes [74]. p53 activation is also causing many of the disease manifestations in the ribosomopathy known as the Treacher Collins syndrome (TCS), a congenital disorder of craniofacial development arising from mutations in the nucleolar protein TCOF1. A deficiency in Tcof1 disrupts ribosome biogenesis and activates p53, and inhibition of p53 prevented neural crest cell apoptosis and rescued the craniofacial abnormalities [75]. 

Taken together, the mouse studies referred to above provide strong experimental support to the notion that p53 has a key role in mediating the phenotypes of the ribosomopathies. Additional support comes from the silencing of ribosomal proteins in zebrafish [76,77] or in cultured mammalian cells that also leads to activation of p53 [51,78,79]. p53 accumulates selectively in isolated primary human hematopoietic progenitor cells after expression of shRNAs targeting *RPS14* or *RPS19,* and the induction of p53 has led to accumulation of p21 and cell cycle arrest specifically in erythroid progenitor cells [80]. The role of p53 in mediating the DBA phenotype is however not entirely clear, as studies in mammalian cancer cell lines show that RPL11 and RPL5 (ribosomal proteins mutated in DBA) are required for p53 activation in response to nucleolar stress [51,78,81]. This in addition to some experimental evidence suggesting that erythroid cell defects in DBA may also in part be p53-independent, as discussed in reference [72]. 

Inhibition of p53 in patients with ribosomopathies should be further explored due to the fact that the phenotypic manifestations of the diseases are mainly p53-dependent. While activation of p53 has long been considered one avenue to effectively target cancer cells, small molecule inhibitors of p53 have been developed and these may now become useful [82]. However, the consequence of long-term p53 inactivation is likely to be an elevated cancer risk. In this regard, inactivation of upstream regulators of p53 for example RPL11, could be one possible way to reduce this risk, keeping p53 functional, able to sense DNA damage or oncogene activation [51]. 

## 7. Mechanisms of p53 Activation by Nucleolar Stress

**Figure 3:**
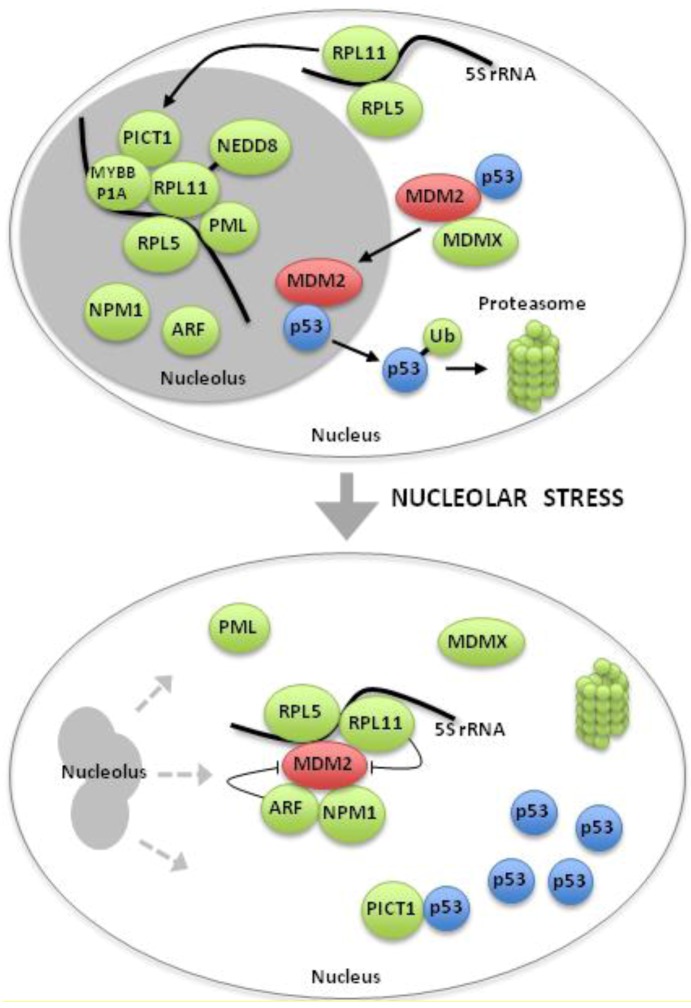
The RPL11-MDM2-p53 molecular network regulating nucleolar stress. Simplified schematic representation of the cell nucleus and nucleolus under normal conditions (upper panel) and following nucleolar stress (lower panel). In the nucleolus, RPL11 is modified by NEDD8 and binds to PICT1 and can also recruit the PML protein. RPL11 and other nucleolar proteins including the histone chaperone NPM1 [136] and the tumor suppressor ARF [137] translocate to the nucleoplasm when the nucleolus is disrupted, and there become increasingly bound to MDM2. As a consequence, MDM2 fails to control the steady state level of p53 that in turn accumulates in the nucleus. Note that there are additional nucleolar and ribosomal proteins that bind to MDM2 and p53 upon nucleolar stress and that they are not shown in the figure. The idea that MDM2 and/or p53 localize to the nucleolus at least transiently is supported by some studies including ref. [85], but the issue remains debated. The exact composition, localization and dynamics of the MDM2/MDMX-5S rRNA-RPL5/RPL11 complex need further investigation.

The mechanism of p53 activation following nucleolar stress is not completely understood. There are at present two main models although they may represent different sides of the same coin [15,25,83,84]. The first model states that p53 is stabilized and activated by default through disruption of normal nucleolar function [29,85], and that the nucleolus itself is required for proper MDM2-dependent degradation of p53. The second model states that nucleolar disruption causes a redistribution of nucleolar and ribosomal proteins to the nucleoplasm, altering their interactions with MDM2 [15]. These re-distributed proteins include ribosomal proteins, p14ARF/p19Arf, nucleostemin, nucleolin and NPM1(B23) among many others (Figure 3.) [86]. The two models will be discussed in more detail below, but it is imperative to point out that translation of p53 mRNA or different post-translational modifications is likely to play a significant role in p53 activation. For instance, acetylation of p53 promotes its transcriptional activity in response to nucleolar stress [87]. A subset of ribosomal proteins has “extra-ribosomal” functions in p53 mRNA translation [88,89]. For example, RPL26 binds to p53 mRNA and is required for its efficient translation in response to irradiation [90]. 

### 7.1. p53 Protein Stabilization By Default

Using micropore irradiation of cell nuclei, Rubbi and Milner could show that extensive DNA damage in the nucleus failed to stabilize p53 unless the nucleolus was also irradiated [29]. Forcing nucleolar disruption by blocking rDNA transcription also resulted in immediate p53 stabilization. In fact, the correlation between nucleolar disruption and abrogation of p53 degradation was evident also after testing other nucleolus disrupting drugs or physical insults. Disturbances in nucleolar function might thus stabilize p53 by preventing its degradation. To investigate this, Rubbi and co-workers designed a novel series of experiments. Based on heterokaryons, photobleaching, and micronucleation, the team demonstrated that p53 degradation is regulated by, and dependent on, an intact nucleolar structure and/or function [85]. One hypothesis designed to explain this result is that the nucleolus is directly involved in nuclear export and/or ubiquitination of p53 [91]. 

### 7.2. Inhibition of the MDM2 E3 Ligase Activity by Ribosomal Proteins

The second model states that a subset of ribosomal proteins binds to the MDM2 protein following disruption of ribosome biogenesis leading to inhibition of the MDM2 E3 ubiquitin ligase activity and p53 stabilization [15]. An early indication that ribosomal proteins could be involved in the regulation of MDM2 came with the report of RPL5 binding to MDM2 in a 5S rRNA-RPL5-MDM2-p53 ribonucleoprotein complex [92]. Subsequently, it was found that the large subunit ribosomal proteins RPL5, RPL11 and RPL23 could bind to MDM2, block the E3 ubiquitin ligase function of MDM2, and promote p53 accumulation [36,93,94,95,96,97,98,99]. In addition, RPS3 [100], RPS7 [101], RPS14 [102], RPS20 [100], RPS25 [103], RPS27 [104,105,106], RPS27-like [104,105,106] and RPL26 [107] have been identified as novel MDM2-binding partners. Interestingly RPL11, but not RPL5 or RPL23, induces accumulation of ubiquitinated MDM2 [108]. This effect is dependent on the ubiquitin ligase activity of MDM2 and requires the central MDM2 binding domain of RPL11 [108]. Here, RPL11 acts through inhibition of 26S proteasome-mediated degradation of ubiquitinated MDM2, thereby prolonging the half-life of MDM2 in cells. Ribosomal protein binding to MDM2 may also play a role in regulating the sub-cellular localization of MDM2 to nucleoli and its interaction with p53 [85]. It should be noted that some ribosomal proteins are targets of the MDM2 E3 ligase including RPL26 [107], RPS27 [106] and RPS7 [101]. RPS7 is a substrate for MDM2 E3 ligase activity both *in vitro* and *in vivo*. An RPS7-ubiquitin fusion protein inhibits MDM2-mediated degradation of p53 and promotes apoptosis to a greater extent than non-Ub-RPS7 [101]. Mutations in the zinc finger of MDM2 were shown to disrupt the binding of RPL5 and RPL11 to MDM2 leading to inactivation of ribosomal protein-mediated inhibition of MDM2 [96,97]. Indeed, a second study confirmed that RPL11 forms a stable complex with MDM2 through direct contact with MDM2´s zinc finger. The study also revealed that the binding between RPL11 and MDM2 is disrupted by single mutations of cysteine as well as non-cysteine amino acids within the zinc finger domain of MDM2, while basic residues in RPL11 are crucial for its stable binding to and suppression of MDM2 activity toward p53 [109]. The physiological significance of the ribosomal protein-MDM2 interaction was strengthened when mice carrying a cysteine-to-phenylalanine substitution in the Mdm2 C4 zinc finger (C305F) were generated [81]. Mdm2^C3^°^5F^ knock-in mice retain a normal p53 response to DNA damage but fail to stabilize p53 protein in response to nucleolar stress most likely because the Mdm2^C3^°^5F^ mutant does not bind RPL11 and RPL5. 

Studies in mice and cell lines show that ribosomal proteins RPL5 and RPL11 are master regulators of MDM2 activity, which underscores that ribosomal proteins play an important role in the p53 pathway. RPL5 and RPL11 are required for p53 to become activated following a deficiency in another ribosomal protein, but knockdown of RPL5 and RPL11 themselves does not significantly activate p53 in mammalian cells cultured *in vitro* [78,81,93,97]. For example, knocking down RPL29 induced p53 leading to cell cycle arrest, whereas p53 activation was inhibited by depletion of RPL11 or RPL5. RPL29 does not bind to MDM2 and does not inhibit MDM2-mediated p53 suppression [110]. It was noted that the level of p53 protein in cells following Actinomycin D treatment is much higher than following knockdown of individual ribosomal proteins from one subunit [79]. RPL23 serves here as an interesting example. RPL23 is required for Actinomycin D-induced p53 stabilization, but knockdown of RPL23 with siRNA on its own induces p53, but to a level that is much lower than in the case of Actinomycin D. The dampening effect on p53 stabilization in cells treated with Actinomycin D by depletion of RPL23 is likely due to a decreased p53 mRNA translation in general, whereas RPL5 and RPL11 directly and specifically in addition inhibits MDM2 to stabilize p53 protein [78]. Please recall that increased p53 mRNA translation may play a role in its activation by cellular stress in many settings and that not only stabilization of the p53 protein is important [78,90]. That RPL11 and RPL5 are different from RPL23 is indirectly supported by the finding that RPL5 and RPL11 binding to MDM2 is disrupted by MDM2 zinc finger mutations, whereas RPL23 can still bind mutant MDM2 [97]. It has also been described that MDM2 itself is able to bind directly to p53 mRNA and thus the possibility that ribosomal proteins may play a role in modulating this process needs to be taken into consideration [111,112]. 

How then is the interaction between MDM2 and RPL11 initiated and maintained? Two models, not necessarily mutually exclusive, have emerged. Previously it was only thought that disruption of the nucleolus promotes a release of RPL11 protein from the nucleolus to the nucleoplasm, but Fumagalli and co-workers found that cells also activate the translation of mRNAs with a polypyrimidine tract at their 5'-transcriptional start site (5'-TOP mRNAs) in response to nucleolar stress [51]. The TOP mRNAs encode among others ribosomal proteins including RPL11. Cells can therefore presumably sustain the levels of free RPL11 available to bind MDM2 in two ways, by releasing a pool of pre-existing nucleolar RPL11 and by boosting its synthesis. It should be noted that nucleolar stress and subsequent growth inhibition might in some settings cause a decline in soluble levels of RPL11 [79,113]. Thus, increased translation of RPL11 mRNA could serve to counteract the destabilization of RPL11 protein following nucleolar stress. In a broader perspective, it was found that depletion of ribosomal proteins results in p53-mediated derepression of microRNA-targeted mRNAs and because microRNAs often repress translation, any changes in expression of ribosomal proteins could have impact on mRNA translation [114].

## 8. Role of Ribosomal Protein-MDM2 Signaling in Cancer and DBA

Sensing nucleolar stress by the building blocks of the ribosome, such as ribosomal proteins themselves, is an interesting example of a tightly balanced regulation of cell growth and cell division. It is possible that the ribosomal protein-MDM2-p53 pathway may have a role in tumor suppression, as indicated by rare MDM2 point mutations in the zinc finger region including C305F seen in patient material [115,116]. Ribosomal protein binding to MDM2 is also of importance to activate p53 in response to oncogenic Myc [81], a master regulator and inducer of ribosome biogenesis and protein synthesis [117]. However there is no overt increase in cancer incidence in MDM2^C3^°^5F^ mice reported to date. By searching the COSMIC database (http://www.sanger.ac.uk/genetics/CGP/cosmic/) for mutations in MDM2, we noticed that point mutations in MDM2 appear infrequent in the analyzed material. Nevertheless, a few of the point mutations reported cluster around the MDM2 zinc finger. Mutations Y281H and W329G were detected in brain and lung cancer, respectively. The MDM2 Y281 residue could possibly be a target for phosphorylation that may alter the zinc finger configuration or affect the binding of ribosomal proteins. W329 is part of the conserved zinc finger but the structural and functional role of this highly conserved residue is not known. In summary, the potential importance of the RPL11-MDM2-p53 pathway in suppression of human tumor development and progression remains unclear and more studies are needed.

As mentioned, another remaining issue is if RPL11 and RPL5 activate p53 in DBA patients. It is conceivable that p53 activation causes at least some of the DBA phenotypes [61,74], but the potential role of p53 in DBA is somewhat confusing given the identification of mutations in RPL5 and RPL11, both of which are required for p53 stabilization in response to nucleolar stress, according to numerous *in vitro* studies. It is possible that on an organism level, a deficiency in RPL5 or RPL11 causes p53 activation by so far unknown mechanisms or could involve the p53-related proteins p63 and p73. Induction of p53 activity by depletion of RPL11 is supported by studies in zebrafish [76], although in zebrafish the role of the RPL11-p53 pathway is presumably different from humans. Moreover, it is generally considered that the activity of p53 is not always related to actual amounts of protein or its stability, so one must carefully assess the activity of p53. Remarkably, many of the mutant ribosomal proteins in DBA and 5q-syndrome are involved in boosting the p53 response, either by translational or post-translational mechanisms (for example: RPS7, RPS14, RPS25, RPS27A, RPL5, RPL11, RPL26). Is it possible that DBA in humans is a consequence of an attenuated p53 response allowing for development into adult life despite defective ribosome biogenesis? Many additional questions remain, and perhaps it may all come down to fine-tuned shifts in p53 activity (either up or down) that will determine the exact manifestation of a ribosome deficiency. In support of the notion that p53 levels are intimately connected to ribosome biogenesis, it was shown by Donati and co-workers that an increase in rRNA synthesis reduces p53 protein levels due to lowered levels of freeribosomal proteins RPL5 and RPL11 [118]. The study revealed a balance between rRNA synthesis, availability of free ribosomal proteins and the level of p53. Such a fine-tuned balance of p53 and ribosome synthesis rate may be involved in influencing the exact disease manifestations of DBA and its related conditions. Some of the discrepancies and unanswered questions are also likely to be explained by p53-independent effects from nucleolar stress.

## 9. Novel regulators of The Ribosomal Protein-MDM2 Complex

Given the importance of ribosomal protein-MDM2 signaling it follows logically that several new regulators of this pathway have been described. These regulators and/or modifiers include MDMX, MYBBP1A, PML, PICT1 and the small ubiquitin related protein NEDD8 among others (Figure 3). **MDMX** is an important regulator of the p53 response to nucleolar stress, as this requires degradation of the MDMX protein in an MDM2-dependent fashion. Cancer cells with abundant MDMX are less sensitive to Actinomycin D due to formation of inactive p53–MDMX complexes. Hence, depletion of MDMX increases sensitivity to Actinomycin D, whereas MDMX overexpression abrogates p53 activation and prevents growth arrest [119]. The promyelocytic leukemia tumor-suppressor protein (**PML**) is known to activate p53 and is involved in regulating MDM2 localization to nucleoli. Interestingly, loss of RPL11 impairs the ability of PML to localize to the nucleoli and regulate MDM2 [120]. Another emerging regulator is an RNA molecule, **5S rRNA**. Marechal and co-workers found that MDM2 binds 5S rRNA, interestingly MDMX also binds 5S rRNA [92,121], and MDMX protein is stabilized by binding to 5S rRNA [121]. In response to nucleolar stress the binding between MDMX and 5S rRNA is disrupted and MDMX is rapidly degraded. The integrated regulation of the MDM2/MDMX-5S rRNA-RPL5/RPL11 complex still remains unknown but could be one key to a more complete understanding of p53 regulation. 

**PICT1** (Protein interacting with the C terminus 1), also known as GLTSCR2 (glioma tumor suppressor candidate region gene 2) is a nucleolar protein, encoded by a gene localized on chromosome 19q13 [122]. PICT1 has emerged as a key regulator of the nucleolar stress response. It was initially described as a tumor suppressor, directly interacting with and stabilizing phosphatase and tensin homolog (PTEN). Low expression of PICT1 in ovarian cancers [123] and diffuse gliomas [124] linked it to tumor malignancy and progression [125]. In agreement with this, enforced expression of PICT1 in glioma cell lines enhanced apoptosis [124]. However, genetic mouse models and murine ES cell models have rather suggested PICT1 as an oncogene. PICT1-/- mice were embryonic lethal as early as at E3.5, whereas PICT1+/- mice developed normally. When investigated in a chemically-induced skin cancer model, PICT1+/- mice were more resistant to develop papillomas compared to PICT+/+ mice [126]. RNAi mediated p53 knock down rescued the mouse ES cells lacking PICT1 from cell cycle arrest although the embryonic lethal PICT1-/- mouse phenotype could not be rescued by combining it with a p53-/- mouse strain. Moreover, introduction of PICT1 RNAi induced p53-dependent growth inhibition in cell lines derived from brain, colorectal and ovarian tumors. 

Using murine ES cells with doxycycline-regulated expression of PICT1, it was shown that PICT1 interacted with RPL11 and sequestered it in the nucleolus. This inhibits the interaction between RPL11 and MDM2 that would otherwise normally take place in the nucleoplasm in response to nucleolar stress. PICT1 overexpression may therefore protect tumor cells from nucleolar stress otherwise resulting in RPL11 mediated p53 stability [126]. Intriguingly, in a separate study by Lee *et al.* it was reported that PICT1 could bind and stabilize p53 in the nucleoplasm [127]. PICT1 translocated to the nucleoplasm from the nucleolus following nucleolar stress and there it prevented MDM2 mediated p53 degradation. Therefore both a deficiency in PICT1, and its overexpression, could lead to p53 activation. This is a situation somewhat similar to what was noted in case of the nucleolar protein nucleostemin. Nucleostemin depletion activates an RPL11-p53 dependent nucleolar stress checkpoint whereas overexpressed nucleostemin binds MDM2 and inhibits p53 degradation [128,129]. Inactivation of the PICT1 yeast ortholog, NOP53p is lethal and Nop53p participates in ribosome biogenesis being required in late rRNA processing events [130]. Therefore loss of a presumed essential function of PICT1 in mammalian ribosome biogenesis may result in nucleolar stress and cell cycle arrest, perhaps independent of RPL11. Different effects from PICT1 could also be explained by alterations in expression level. Endogenous levels or marginally elevated levels of PICT1 may promote RPL11 nucleolar localization whereas high expression of PICT1 in addition could “spill over” to the nucleoplasm and bind to p53. 

The ubiquitin like molecule **NEDD8** (neural-precursor-cell-expressed developmentally down-regulated 8) a 9 kDa 81-amino acid protein, modifies its substrates through an E3 ligase dependant process called neddylation [131]. This process is highly relevant in the regulation of RPL11-MDM2 binding. NEDD8, like ubiquitin, is first synthesized as a precursor and then processed through a conjugation cascade involving E1 activating (AppBp1/UBA3 or NAE) and E2 conjugating (UBC12, UBE2F) enzymes followed by E3 ligation (neddylation) to substrates. NEDD8 neddylation is a reversible process, in which the COP9 signalosome, NEDP1/DEN1/SENP1 and USP1 can remove NEDD8 from its substrates. The NEDD8 pathway plays an important role in activating the ubiquitin E3 ligase activity of CRL E3s (cullin-RING ligases) via the covalent attachment of NEDD8 to the core cullin protein of these enzyme complexes [132]. CRLs are responsible for ubiquitinating a number of substrate proteins essential for cellular functions implicated in cancer and are overexpressed, amplified and mutated in several human cancers. Neddylation is furthermore linked to cancer pathways by promoting MDM2 stability and MDM2 E3 ligase activity which neddylates p53 [133,134] thereby affecting p53 gene target specificity [135]. MDM2 also neddylates RPL11, a process that can take place in the cytoplasm, protecting RPL11 from degradation and leading to its enhanced nucleolar localization [113]. Upon nucleolar stress there is an induction of RPL11 de-neddylation by NEDP1 [113], leading to nucleoplasmic localization of RPL11. In the nucleoplasm RPL11 (like other ribosomal proteins) is targeted for proteasomal degradation, but also free to interact with MDM2 [113]. Thus, nucleolar stress can induce an RPL11 mediated p53 response that is dependent on NEDD8. Mahata *et al.* found that reduced RPL11-NEDDylation due to nucleolar stress, allows recruitment of RPL11 at p53 regulated promoters and RPL11 enhances recruitment of p53 transcriptional co-activators (CBP/p300) [135]. Hence, RPL11 blocks both MDM2 mediated degradation of p53 as well as enhances p53´s transcriptional activity on chromatin.

As mentioned, acetylation of p53 may play an important role in stabilization of the protein and activation of its transcriptional functions. As recently described, Myb-binding protein 1a (**MYBBP1A**) is involved in p53 acetylation and stabilization [87]. MYBBP1A was originally identified as a c-myb proto-oncogene product (c-Myb)-interacting protein that localizes to nucleoli requiring rRNA and MYBBP1A associates with both RNA polymerase I complex and pre-ribosomes. When rRNA transcription is suppressed by nucleolar stress, MYBBP1A translocates to the nucleoplasm and facilitates p53-p300 interaction to enhance p53 acetylation [87]. RPL5 or RPL11 depletion inhibits MYBBP1A translocation and p53 activation, since rRNA/pre-ribosomes are not properly exported from the nucleolus when cells are lacking RPL11/RPL5 [87]. This study raises some interesting questions as with regard to the mechanism(s) whereby depletion of RPL11/RPL5 impairs the p53 nucleolar stress response. Perhaps it is not only binding to and inhibition of MDM2 E3 ligase activity that matters?

## 10. p53-Independent Nucleolar Stress Pathways

The consequences of nucleolar stress in p53 wild type cells are complex. However, it is often so that cancer cells contain mutant p53 or no p53 at all, so how does the cell cycle machinery respond to nucleolar stress in these cells? It has been observed that nucleolar stress in HeLa cells (that are considered to lack functional p53 due to viral inactivation) triggered cell cycle arrest and/or apoptosis [79,114]. Moreover, it was found that inhibition of RNA pol I in p53-/- cells resulted in decreased expression of the E2F-1 transcription factor due to release of RPL11, that in turn binds MDM2 and inactivates the E2F-1 stabilizing function ascribed to MDM2 [47]. This study revealed the existence of a p53-independent, but still RPL11-dependent, mechanism that links nucleolar stress to cell cycle arrest. As another example, it was shown that nucleolar stress destabilizes the proto-oncogene serine/threonine-protein kinase PIM1 causing the levels of p27Kip1 to increase, and this in turn contributes to the cell cycle arrest seen in p53-/- cells [138]. It will be of interest to investigate the role of E2F-1 and PIM1 kinase down regulation in the DBA phenotypes. Two related p53 proteins, p63 and p73, so far not implicated in nucleolar stress must be considered as well, especially given their prominent role in organism development [139]. Finally, it is known that MDM2 has many p53-independent effects in cells besides controlling E2F1 and p53 [140]. These functions could be affected by changes in ribosomal protein binding as well.

RPL11 does not only serve as a master regulator of MDM2 and p53, but RPL11 in addition regulates c-Myc mRNA turnover and c-Myc protein activity. RPL11 may inhibit c-Myc activity by blocking the recruitment of its co-activator TRRAP to the promoter regions of c-Myc target genes that are transcribed by RNA polymerases I/II [141], and 5S rRNA/tRNA genes by RNA polymerase III (also Myc targets) [142]. In response to nucleolar stress, RPL11 binding to these genes was increased, and inversely TRRAP binding was decreased. As previously mentioned, RPL11 is recruited at promoter sites of p53-regulated genes upon nucleolar stress [135]. In addition, RPL11 binds to c-Myc mRNA at its 3' untranslated region (3'-UTR) leading to c-Myc mRNA reduction. Nucleolar stress decreases the c-Myc mRNA levels in an RPL11-dependent manner [143]. These studies taken together indicate extra-ribosomal RPL11 functions likely to be of importance in the nucleolar stress response. These unexpected functions of a ribosomal protein (RPL11) would be in agreement with the notion that ribosomal proteins may have “extra-ribosomal” functions in gene regulation [86,144,145]. Since c-Myc is known as a master regulator and inducer of ribosome biogenesis/protein synthesis these exciting data place RPL11 in a context where it can oversee and modify the entire process of ribosome biogenesis and ultimately cell growth. 

## 11. Conclusions

Studies in mice bearing conditional deletions of ribosomal protein genes [58] and the discoveries of mutated ribosomal proteins in various ribosomopathies [66] revealed that mammalian cells have a surveillance system for monitoring ribosome biogenesis and that p53 is activated if errors in this process are encountered. Many studies also demonstrate that the nucleolus itself serves as a stress-response organelle for several types of damaging insults. Elucidating the mechanisms of nucleolar stress is of importance to understand the full effects and potentials of anti-cancer treatments as well as the pathogenic mechanisms of the ribosomopathies. The emerging two main nucleolar stress mechanisms, activation of p53 and degradation of E2F-1, are suggested to be key events in cells linking a defective ribosome biogenesis to a stop in the cell cycle. While the role of the ribosomal protein-MDM2-p53 pathway in connecting ribosome biogenesis to the cell cycle machinery is becoming more accepted, its putative broader function in cell metabolism remains to be further explored. The function of RPL11 in regulating c-Myc function deserves more attention and represents a third and potentially very important nucleolar stress outcome.

Ribosome biogenesis in malignant cells should be exploited as an anti-cancer therapy target since this is one of the major biosynthetic activities in the cancer cell [146]. In particular, p53 activation by nucleolar stress could be used to our advantage as shown in treatment of B-cell lymphoma in mice [46]. Blocking RNA pol I could become effective in p53 deficient tumors as well, since nucleolar stress induces degradation of E2F-1 and inhibition of c-Myc. The pathways of nucleolar stress are however becoming more and more complex and there is a need to further characterize the molecular mechanisms involved to safely modify them. One thing that in our opinion needs to be addressed in anti-cancer treatment is to measure the clinical benefit of selectively targeting ribosomal biogenesis. However, to dissect clinical efficacy of the different *in vivo* responses (nucleolar stress response *vs* DNA damage response) is not trivial given partially overlapping effects and variable concentrations of drugs within a tumor. The MDM2^C3^°^5F^ mouse model could shed further light on this issue, since these mice retain the normal DNA damage response but fail to stabilize p53 due to nucleolar stress [81]. A *caveat* could be that other yet unknown MDM2 functions may be affected by this mutation and confound the analysis.

So why then, is p53 activated following nucleolar stress? One explanation is that p53 induction allows the cell to correct for an unbalanced or defective ribosome synthesis before cell division [84]. p53 could accomplish this by suppression of RNA pol I activity [147] and/or by inducing cell cycle arrest. On the down side, active p53 mediates many of the negative clinical phenotypes in the ribosomopathies. As mentioned, inactivation of p53 has been proposed as a relevant therapeutic strategy for treating these diseases [73]. The functions of p53 in cancer, ageing, and metabolism are numerous and complex with close ties to ribosomal proteins, nevertheless the manipulation of these functions may hold key insights for future treatments against ribosomopathies and cancer, but needs careful analysis. However, one must also consider that a defective ribosome biogenesis could have serious adverse effects in the long run. Non-lethal ribosome abnormalities, if kept unattended, may lead to changes in mRNA translation patterns or cause changes in DNA regulation, perhaps resulting in DNA damage and ultimately increased risk of cancer.
